# Five draft genome assemblies from Bacillaceae isolated from a degraded wetland environment

**DOI:** 10.1128/mra.00845-23

**Published:** 2023-12-22

**Authors:** Anna L. McLoon, Prince Ackaah Asante, Thomas Anderson, Kellyanne Cahill, Delana Cochrane, Keira Cohen, Jaylene German, Christian M. Hrubes, Isabella LaCroix, Killian McNamee, Anna Mossakowski, Aidan M. Nichter, Jessica L. Pepe, Andrew T. Schofield

**Affiliations:** 1 Department of Biology, Siena College, Loudonville, New York, USA; Wellesley College, Wellesley, Massachusetts, USA

**Keywords:** genomes, Bacillaceae, wetlands

## Abstract

We isolated five Bacillaceae from a degraded wetland environment and sequenced their genomes using Illumina NextSeq. Here, we report draft genome sequences of *Bacillus velezensus-*SC119, *Priestia megaterium* strain SC120, *Bacillus zhangzhouensis* strain SC123, *Bacillus pumilis* strain SC124, and *Bacillus idriensis* strain SC127. The genomes range between 3,657,353 and 5,772,725 bp with % GC between 37.62% and 46.38%.

## ANNOUNCEMENT

Wetland environments play critical roles in the terrestrial carbon and water cycles, and microbial communities are key players in ecosystem function (1). Endospore-forming bacteria in the Bacillaceae family are metabolically and genomically diverse soil heterotrophs that influence plant health and nutrient cycling, and produce diverse natural products that influence bacterial and non-bacterial species in their environment (2).

We collected two 30-mL soil samples on 19 January 2023 from 42°43′7″N, 73°45′01.4″W. One was highly hydrated and within a patch of invasive common reeds (*Phragmites* sp.), and the other was near the base of an Eastern cottonwood tree (*Populus deltoides*). *Bacillus pumilis* strain SC124 was isolated from the soil from near the tree, while *Bacillus velezensis* strain SC119, *Priestia megaterium* strain SC120, *Bacillus zhangzhouensis* strain SC123, and *Bacillus idriensis* strain SC127 were isolated from the marshy soil (Table 1). Unique bacterial strains were isolated as described previously by boiling 100- to 400-μL soil subsamples for 10 minutes in 1-mL water, and suspended cells were spread by swab on Tryptic soy agar (TSA) + 5% sheep blood and incubated for 24 hours at 37°C (3). All strains grew and remained viable at temperatures between 22°C and 37°C. After colony purification and basic characterization, genomic DNA was isolated and sequenced as described previously (3) using a Promega DNA wizard kit from cultures grown for approximately 3 hours at 37°C in tryptic soy broth, and libraries were prepared and sequenced as 151-bp paired-end reads using an Illumina NextSeq 2000 instrument by SeqCenter, which prepared libraries using the Illumina DNA Prep kit and IDT 10-bp UDI indices (Pittsburgh, PA). Demultiplexing, quality control, and adapter trimming were performed with bcl-convert v.3.9.3 (Illumina). Sequence reads were imported into the KBase environment for analysis, with each genome analysis occurring in parallel in its own narrative (4–6). Read quality was checked with FastQC v.0.11.09; reads were trimmed with Trimmomatic v.0.36, assembled *de novo* using SPAdes v.3.15.3, and annotated using RASTtk v.1.073 and Prokka v.1.14.5; probable species identities were determined using both GTDB-Tk v.1.7.0 and TYGS, which were in agreement in all cases; and metabolic predictions were made using DRAM v.0.1.2 (7–20). Default parameters were used when running all programs.

**TABLE 1:** Summary of genome statistics and access

Strain	SC119	SC120	SC123	SC124	SC127
Species	*Bacillus velezensis*	*Priestia megaterium*	*Bacillus zhangzhouensis*	*Bacillus pumilus*	*Bacillus idriensis*
Number of reads	7,568,064	8,044,838	6,292,516	6,681,492	6,671,362
Number of contigs	23	27	22	21	15
Total length (bp)	3,947,238	5,772,725	3,657,353	3,837,327	4,483,053
N50	578,987	4,216,129	339,228	824,075	675,715
% GC	46.38	37.62	41.43	41.33	41.01
Predicted genes (Prokka via Kbase)	3,875	6,010	3,713	3,981	4,498
Predicted genes (GenBank)	3,908	6,013	3,722	3,963	4,492
KBase narrative	https://kbase.us/n/139243/37/	https://kbase.us/n/139247/20/	https://kbase.us/n/139245/53/	https://kbase.us/n/139244/41/	https://kbase.us/n/139250/65/
SRA accession	SAMN37195270	SAMN37195271	SAMN37195272	SAMN37195273	SAMN37195274
GenBank accession	JAVIKC000000000	JAVIKB000000000	JAVIKA000000000	JAVIJZ000000000	JAVIJY000000000

Surprisingly, despite strain isolation from boiled soil samples and genes necessary for endospore formation, isolates showed dramatic differences in sporulation following 24-hour 37°C incubation on Difco Sporulation Medium (DSM), 0.8% Difco nutrient broth, 0.1% KCl, 0.0012% MgSO_4_, 1% agar, and pH 7.6) plates (21); *B. velezensis* strain SC119 achieved high sporulation efficiency, measured by outgrowth of boiled and unboiled serial dilutions, while *B. idriensis* strain SC127 did not detectably sporulate. These strains and their genomes provide additional insights into the genetic basis for phenotypic variation between closely related species (Table 1).

## Data Availability

Genome sequences and raw sequencing reads were deposited under BioProject accession number PRJNA862062, Sequence Read Archive accession numbers SAMN37195270, SAMN37195271, SAMN37195272, SAMN37195273, and SAMN37195274 and GenBank genome accession numbers JAVIKC000000000, JAVIKB000000000, JAVIKA000000000, JAVIJZ000000000, and JAVIJY000000000. Analyses are available through KBase narratives https://kbase.us/n/139243/37/, https://kbase.us/n/139247/20/, https://kbase.us/n/139245/53/, https://kbase.us/n/139244/41/, and https://kbase.us/n/139250/65/, and the overview is narrative at https://kbase.us/n/152701/18/.
